# Comprehensive Risk Assessment of Health-Related Hazardous Events in the Drinking Water Supply System from Source to Tap in Gaza Strip, Palestine

**DOI:** 10.1155/2020/7194780

**Published:** 2020-01-29

**Authors:** Samer Abuzerr, Mahdi Hadi, Kate Zinszer, Simin Nasseri, Masud Yunesian, Amir Hossein Mahvi, Ramin Nabizadeh, Shimels Hussien Mohammed

**Affiliations:** ^1^Department of Environmental Health Engineering, School of Public Health, International Campus, Tehran University of Medical Sciences, Tehran, Iran; ^2^School of Public Health, Department of Social and Preventive Medicine, University of Montreal, Montréal, Canada; ^3^Quality Improvement and Infection Control Unit, Ministry of Health, Gaza, State of Palestine; ^4^Center for Water Quality Research (CWQR), Institute for Environmental Research (IER), Tehran University of Medical Sciences, Tehran, Iran; ^5^Department of Environmental Health Engineering, School of Public Health, Tehran University of Medical Sciences, Tehran, Iran; ^6^Department of Research Methodology and Data Analysis, Institute for Environmental Research, TUMS, Tehran, Iran; ^7^Department of Community Nutrition, School of Nutritional Sciences and Dietetics, Tehran University of Medical Sciences, Tehran, Iran

## Abstract

**Background:**

The traditional approach in the management of the quality drinking water, and relying on end-product testing, has proven ineffective in protecting public health. Therefore, the transition to a systematic approach in drinking water supply systems management from the source to the consumer tap was taken as a water safety plan (WSP).

**Objective:**

The study aims to investigate the health-related hazardous events in order to decide on the best risk-reduction strategies in the supply of drinking water in the Gaza strip.

**Methods:**

A semiquantitative matrix method for risk assessment was applied. Also, chlorine residual, electrical conductivity, and nitrate concentration further tested in 109 water wells, 109 small-scale water desalination plants, 197 tanker trucks, and 384 households distributed over five governorates of the Gaza strip.

**Results:**

The mean of the measured chlorine residual values was less than the recommended national and international limits (0.2–1 mg/liter). The mean of electrical conductivity at catchment points and household municipal water taps was 2165.1 *μ*S·cm^−1^ and 2000 *μ*S·cm^−1^, respectively. Furthermore, zero percent of water samples met the recommended criteria, indicating that the groundwater in the Gaza strip is nonpotable. Only 12.8% and 8.8% of water samples met the permissible levels at catchment areas and municipal water at household, respectively, indicating sever health impacts on the public. Moreover, the most hazardous events were related to high levels of groundwater salinity, the low level of disinfection, the effect of electricity outages on the efficiency of the desalination process, and leakage of water from the tanker truck tank reservoirs. Therefore, urgent interventions are required to improve the quality of water and to mitigate the possible health effects.

**Conclusion:**

The prioritization of hazardous events that are proportional to the degree of their attributed risk could help guide in making the right risk-reduction decisions. Urgent interventions are required to improve the quality of water and to mitigate the possible health effects.

## 1. Introduction

Possessing safe drinking water is crucial for the development of sustainable health and a booming economy for any society [1, 2]. Despite the efforts of drinking water supply agencies around the world to provide safe drinking water, waterborne diseases cause 2.4 million deaths and 73 million disability-adjusted life years (DALYs) [3, 4]. Furthermore, natural and anthropogenic threats to the drinking water supply systems (DWSS) in urban areas lead to adverse human health outcomes [5, 6]. Therefore, the full control of the drinking water supply systems considering the surrounding environmental factors has a vital role in reducing the risk of potential contamination [7].

The conventional approach of water supply management, relying on end-product testing, has proven to be ineffective in protecting public health and the quality of drinking water. Hence, a transition to an integrated risk management approach for the management of DWSS from the catchment to the consumer tap was adopted at the beginning of 2004 [8]. The water safety plan (WSP) was introduced in the third edition of the World Health Organization's guidelines for drinking water quality (GDWQ) as a useful tool to systematically assess and manage risks in DWSSs from source to consumption point [1, 9–11].

More recently, in the WSP workshop in Berlin in 2014 for the assurance provision of safe drinking water to citizens in the twenty-first century, the WSP also was approved as a systematic and valuable managerial tool [12]. Moreover, several countries have been welcomed the adoption of the WSP as national legislation to achieve outstanding successes in managing DWSSs [9, 13–15].

The applied risk assessment approach in this study was relied on using a semiquantitative matrix method. In this approach, the risk was quantified based on the probability of hazardous events occurrence (due to exposure with biological, physical, chemical, or radiological agents) and the severity of their consequences on the DWSSs [16, 17].

DWSSs in the Gaza strip, as in developing countries, are vulnerable to different kinds of hazards. The deterioration of groundwater quality, the only source of water in the Gaza strip, has prompted the establishment of brackish water small-scale desalination plants as a strategic solution to meet the community needs for potable water. Nowadays, the vast majority of Gaza's population relies on desalinated water, mainly for drinking purposes. However, the problems related to water quality due to the presence of microbial, chemical, and physical agents have been reported in Gaza's DWSSs, primarily due to the nonhygienic practices during water transportation or storage [18–22]. Moreover, we underline the fragile state of coordination among institutions in charge of the water sector in the Gaza strip [23].

To the best of our knowledge, this is the first study that provided an overarching description of most critical hazardous events in drinking water supply systems from source to tap in the Gaza strip in order to enable water supply providers and stakeholders to decide on the best risk-reduction strategies.

## 2. Materials and Methods

### 2.1. Study Setting

**Figure 1:**
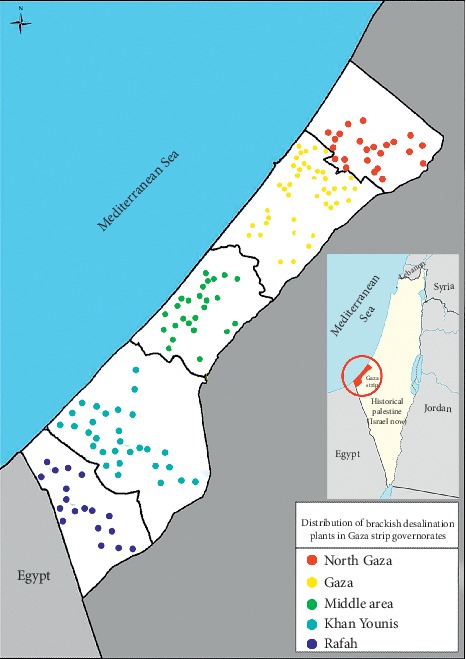
Gaza strip map and distribution of small-scale water desalination plants in the five governorates.

The current study was conducted in the Gaza strip between March 2018 and July 2018. This region is the southwestern corner of Palestine with an area of around 365 km^2^. It shares borders with the lands of historical Palestine (so-called Israel now) to the east and north, Egypt to the south, and the Mediterranean Sea to the west. Its population was estimated at 1,912,267 inhabitants distributed over five governorates with high population density at 5,324 persons/km^2^ (Figure 1) [24].

### 2.2. Study Design and Sampling

**Figure 2:**
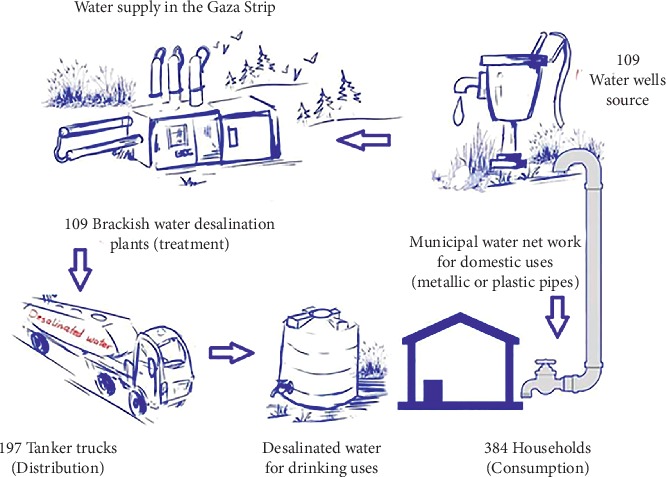
Schematic illustration map of a typical Gaza strip's water supply systems.

An observational study was carried out from March 2018 to July 2018 to identify the hazardous events in each part of a typical drinking water supply system in the Gaza strip, including water wells, desalination plants, tanker trucks, and municipal water and desalinated water at households. It is worth mentioning that there are two water supply systems in the Gaza strip. The first is for nondesalinated municipal water for domestic purposes, whereas the second is for desalinated water for drinking purposes (Figure 2).

More than 150 small-scale brackish desalination plants exist along the Gaza strip in order to improve the quality of groundwater [20]. A cluster sampling was adopted in our study, as the study sample was distributed into five governorates based on the population density in each governorate.

Seven experts in the fields of water resource management, environmental health, and public health were recruited as WSP team members. They underwent a five-day training to enable them define and identify adequately the likelihood of occurrence and severity of each hazardous event and avoid subjective assessment during field visits. The training package is inspired by the “Drinking Water Safety Plan Training Course,” which is sponsored by Alberta Environment and Sustainable Resource Development. The training was offered by the study authors [25].

The WSP team members joined the field visits together, and each of them reported the likelihood of occurrence and severity of each hazardous event individually on his datasheet, and then, the average of the reported likelihoods and severity values was considered.

Hazards in this study refer to biological, chemical, and physical agents. Approximately 109 water wells, 109 small-scale water desalination plants, 197 tanker trucks, and 384 households were included in this study.

### 2.3. Study Tools

#### 2.3.1. Water Testing

Three field-based chemical water quality tests including residual chlorine, electrical conductivity, and nitrate ions level were tested. The tests were conducted in triplicates, and the mean values were recorded. The residual chlorine was measured according to the standard colorimetric method using the N,N′-diethyl-p-phenylenediamine (DPD) reagent [26].

Electrical conductivity was measured using a portable instrument called a DDS 307 conductivity meter. Nitrate was measured by aquaread nitrate meter for water testing [27].

#### 2.3.2. Risk Matrix

A matrix of five rows and five columns was used as an instrument for observation and assessment. The columns represent the degree of likelihood of the hazard occurrence, whereas the rows denote the severity of the hazard occurring. Each hazardous event was rated individually and after that combined into an overall risk score [16].

Value for the likelihood of the occurrence of each undesirable event is defined in Table 1 [1]. The risk matrix of the catchment, small-scale desalination plant, tanker trucks, and households included 14, 20, 18, and 18 potential hazardous events, respectively.

### 2.4. Hazardous Events Identification

The hazardous events of each risk matrix were developed after reviewing the relevant scientific and technical information to match with the local context of each drinking water supply system in the Gaza strip. The WSP team independently validated the final draft of the matrix.

### 2.5. Sample Size Determination

The study population comprised 260 municipal water wells, 150 small-scale brackish desalination plants, 400 tanker trucks, and 303,330 households distributed over the five governorates of the Gaza strip. The sample size was calculated using Epi Info program with a margin of error of 5%, confidence level of 95%, and a response distribution of 50%. Hence, the calculated sample size was 109 municipal water wells, 109 small-scale brackish desalination plants, 197 tanker trucks, and 384 households.

### 2.6. Data Analysis

Each completed matrix was manually checked in order to ensure the quality of the collected data in the matter of completeness, clarity, and uniformity before it could be coded in MS Excel 2007. The mean, standard deviation, minimum, maximum, and percentage of samples that met the recommended limits were calculated for the readings of chlorine residual, electrical conductivity, and nitrate throughout the DWSSs.

The average and standard deviation were calculated as well as for the likelihood, severity, and degree of risk for each hazardous event.

## 3. Results

### 3.1. Water Chemistry Parameters

Interestingly, the mean of the measured chlorine residual values in DWSSs was less than the recommended limits of both Palestinian water authority (PWA) and world health organization (WHO) (0.2–1 mg/liter). We found decreased residual chlorine levels in both catchment areas and the households. The following are the percentage values of the various sites that met the recommended chlorine residual criteria: catchment areas 31.2%, treatment plants 18.3%, tanker trucks 14.7%, desalinated water at households 4.3%, and municipal water for households 30.2%. A marked decrease in electrical conductivity before and after the desalination process was recorded. The mean of electrical conductivity at catchment points and at household municipal water taps was 2165.1 *μ*S·cm^−1^ and 2000 *μ*S·cm^−1^, respectively.

Furthermore, zero percent of water samples met the recommended criteria (250 *μ*S/cm), indicating that the groundwater in Gaza strip is nonpotable water and generally contains a high level of dissolved solids. Despite acceptable nitrate levels in the desalinated water, high levels of nitrates were recorded in the DWSSs, and only 12.8% and 8.8% of water samples met the PWA and WHO permissible levels (70 and 50 mg/L, respectively) at the catchment areas and municipal water at households, respectively, indicating severe health impacts on public health (Table 2).

### 3.2. Assessment of Hazardous Events

The assessment of health-threatening hazardous events in DWSSs using inspection forms helps in providing a comprehensive and integrated understanding of risks and their control measures [28]. Risk characterization was provided in the context of the multiple-barrier approaches, so it categorizes each hazard by its associated barrier. For instance, a pipeline leakage is a hazardous event associated with the water distribution system and maintenance barrier. Consequently, linking risks to barriers facilitates an analysis of the effectiveness and reliability of protective barriers. The hazardous events were arranged in descending order according to the calculated degree of risk for each hazardous event in DWSSs.

#### 3.2.1. Catchment Points

At water storage tanks of abstracted groundwater, the high level of salinity of groundwater had the highest degree of risk, followed by excessive algal formations. However, nine hazardous events were found to cause a medium degree of risk, whereas two hazardous events formed a low degree of risk (Table 3).

#### 3.2.2. Production Points

At brackish small-scale desalination plants, the highest degree of risk was due to the low level of chlorination and because of the effect of power outages on the efficiency of the desalination process. One hazardous event was found to cause a medium degree of risk, which is the mixing of desalinated water with nondesalinated water in water storage tanks. Nevertheless, the rest of the hazardous events constituted a low degree of risk (Table 4).

#### 3.2.3. Transportation Points

Tanker trucks posed a very high degree of risk caused by the low level of disinfection, the excessive algal formations inside the tanker truck reservoir, and leakage of water. Whereas a medium degree of risk as a result of lack of tanker truck license by the Ministry of Health and the absence of drainage tap at the bottom of the reservoir. The other hazardous events resulted in a low degree of risk (Table 5).

#### 3.2.4. Consumption Points

At households, a very high degree of risk was due to the low level of disinfection. A high degree of risk was because of the excessive algal formations inside the drinking water storage tanks. A medium degree of risk as a result of negligence of the periodic washing of drinking water tanks with chlorine and soap. The other hazardous events led to a low degree of risk (Table 6).

## 4. Discussion

Despite the persistent efforts to overcome the DWSSs problems in the Gaza strip, as in most developing countries, the Gaza strip is still suffering from the presence of water quality challenges.

The results of this study highlighted the most hazardous events in the drinking water supply system in the Gaza strip, which are mainly related to the high levels of groundwater salinity, the low level of disinfection, the growth of biofilm bacteria inside water storage tanks, the effect of electricity outages on the efficiency of the desalination process, and leakage of water from the tanker truck reservoirs. Several studies have been conducted to assess chemical, biological, and physical hazardous events individually in Gaza's water supply systems [29–31].

The high level of aquifer salinity was attributable to the mobilization of deep brines and seawater intrusion due to overabstraction of groundwater [32–34]. Several recommendations were proposed by Abbas et al. and Abuzerr et al. aimed to improve the quality of groundwater as well as the areas surrounding the water wells [35, 36].

Because we noticed that water desalination plants apply chlorination before the desalination process, not after, preceding reports have proven a significant correlation between the prevalence of waterborne diseases and inadequate chlorination of drinking water in Gaza [18, 37–39]. Moreover, Sadallah and Al-Najar study showed a significant association between the high incidence of diarrheal diseases among the population of Um Al Nasser village and the low level of residual chlorine in drinking water networks [29].

The results of our study also showed a gradual decrease in residual chlorine levels with the progress in DWSS parts, starting from the source point and ending with consumption point at household. We can state that Gaza's DWSS, in general, suffered from inadequate disinfection, which increases the risk of biofilm formation and increases the chance of bacteria regrowth [40]. These outcomes are consistent with Aish, 2013 findings, which revealed an increase in microbiological contamination level throughout Gaza's DWSSs [30]. It is worth mentioning that we have noticed that many desalination plants were applying chlorination before the desalination process and not after. Also, the owners of brackish desalination plants do not add sufficient amounts of chlorine to the desalinated water due to aesthetical issues such as odor and taste issues because they claim that Gazans do not like the taste of chlorine in drinking water, so they try to keep their customers satisfied. Nevertheless, in order to protect the public and to ensure the safety of DWSSs, the PWA has adopted the WHO chlorine residual limit as a national standard [41]. Accordingly, constant monitoring of chlorine residual level in public drinking water supply systems is crucial [42–44].

Surprisingly, we found an increase in the electrical conductivity level in the desalinated water at desalination plants in comparison with the desalinated water at households (Table 2).

This could probably be attributed to the erroneous practices of mixing the nonimproved water with desalinated water. Moreover, from our point of view, the corrupt practices of waters mixing at desalination plants and tanker trucks mainly takes place due to shortage of electricity supplies in the Gaza strip. This situation prevents the operation of desalination plants for more than 8 hours a day, thereby compelling the owners of desalination plants and tanker trucks to mix the waters in order to meet customers' demands. Waters mixing at the household might happen because of the inability of a household to procure desalinated water. Accordingly, the introduction of a cross-subsidy technique to support poor households that cannot afford the WHO limit of 3 New Israeli Shekel per cubic meter (NIS/m^3^) (1 USD ≈ 3.5 NIS). Besides, seeking sustainable cost-effective energy alternatives to increase the efficiency of the desalination plants is highly recommended [45, 46]. The high levels of nitrate ions we found in this study are consistent with the previous investigations carried out in the Gaza strip that linked its source to improper wastewater disposal and overuse of chemical fertilizers in the agricultural activities [32, 47].

## 5. Conclusion and Recommendation

In this study, the prioritization of hazardous events in DWSSs according to their degree of risk was carried out to decide on the best risk-reduction strategies. This study demonstrated the inadequacy of chlorination in Gaza's DWSSs. The risk levels of hazardous events were ranged from low to very high. The highest levels of risks were related to the high salinity level of groundwater, the low level of disinfection, the formation of biofilm bacteria inside of drinking water storage tanks, the effect of electricity outages on the efficiency of the desalination process, and leakage of water from the tanker truck reservoir. Surprisingly, we found an increase in the electrical conductivity level in the desalinated water at desalination plants in comparison with the desalinated water at households. This was attributed to the unfortunate practice of mixing the nonimproved water with desalinated water. High levels of nitrate were found in the DWSSs, indicating sever health impacts on the public. Therefore, in view of these findings, we recommend further efforts and application of more control measures to reduce the risk of hazardous events on DWSSs as well as to provide safe drinking water for the community following the Palestinian water authority guidelines.

## Figures and Tables

**Table 1:** Semiquantitative risk analysis matrix.

		Severity				
		Insignificant or no impact—rating: 1	Minor compliance impact—rating: 2	Moderate aesthetic impact—rating: 3	Major regulatory impact—rating: 4	Catastrophic public health impact—rating: 5
Likelihood or frequency	Almost certain/once a day—rating: 5	5	10	15	20	25
	Likely/once a week—rating: 4	4	8	12	16	20
	Moderate/once a month—rating: 3	3	6	9	12	15
	Unlikely/once a year—rating: 2	2	4	6	8	10
	Rare/once every 5 years—rating: 1	1	2	3	4	5
Risk score		<6	6–9	10–15		>15
Risk rating		Low	Medium	High		Very high

**Table 2:** Chlorine residual, electrical conductivity, and nitrate measurements.

	Chlorine residual (mg/liter)				Electrical conductivity (*μ*S/cm)				Nitrate NO^3−^ (mg/L)			
Local standard	PWA		0.2–1		PWA		250		PWA		70	

International standard	WHO		0.2–1		WHO		250		WHO		50	

DWSS	Mean ± SD	Min	Max	Samples met the standard (%)	Mean ± SD	Min	Max	Samples met the standard (%)	Mean ± SD	Min	Max	Samples met the standard (%)

Catchment (*n* = 109)	0.19 ± 0.3	0	0.9	31.2	2165.1 ± 969.8	750	5000	0	180.4 ± 108.9	35	445	12.8

Treatment plant (*n* = 109)	0.09 ± 0.2	0	0.9	18.3	223.1 ± 162.6	60	1010	71.5	18.8 ± 12.9	1.32	60	100

Tanker truck (*n* = 197)	0.03 ± 0.1	0	0.9	14.7	226.4 ± 187.6	50	1200	70.7	19.9 ± 12.9	1.32	64	100

Desalinated water at household (*n* = 384)	0.01 ± 0.06	0	0.3	4.3	235.4 ± 186.4	50	1300	41.9	21.9 ± 13.3	4.91	68	100

Municipal water at household (*n* = 384)	0.19 ± 0.3	0	0.9	30.2	2000.3 ± 908.5	750	5000	0	202.6 ± 11.02	43	456	8.8

**Table 3:** Assessment of hazardous events at catchments by seven raters.

Hazard	Hazardous event	Likelihood avg (SD)	Severity avg (SD)	Risk level avg (SD)
Chemical	High level of dissolved salts in water	4.11 (0.65)	3.41 (0.58)	14.04 (3.26) (high risk)

Microbial and chemical	Excessive algal formations in water storage tanks	2.93 (0.52)	3.40 (0.66)	10.15 (3.19) (high risk)

Microbial	Sewage collection within 200 m of the borehole	2.38 (0.73)	2.97 (0.79)	7.48 (3.76) (medium risk)

Microbial	Water leakage between water well and water storage tank	2.54 (0.63)	2.82 (0.63)	7.41 (3.21) (medium risk)

Microbial	The leaking water forms stagnant ponding within 10 m of the borehole	2.49 (0.74)	2.69 (0.75)	7.09 (3.50) (medium risk)

Chemical and microbial	Spilled water drainage channels are absent or cracked or unclean	2.40 (0.70)	2.67 (0.73)	6.77 (3.44) (medium risk)

Microbial	Human, animals, or birds excreta on the ground within 10 m of the borehole	2.25 (0.66)	2.80 (0.75)	6.62 (3.29) (medium risk)

Microbial	The fence of the water well is inadequate to prevent the entry of animals	2.36 (0.64)	2.68 (0.66)	6.61 (3.10) (medium risk)

Chemical	Accidental spillage of diesel or other chemicals within 100 m of the borehole	2.33 (0.65)	2.68 (0.76)	6.60 (3.30) (medium risk)

Chemical, microbial, and physical	The cement cap of a borehole is absent, cracked or unclean	2.33 (0.66)	2.57 (0.69)	6.29 (2.99) (medium risk)

Chemical and microbial	The pump house at the point of attachment to the borehole base is loose	2.28 (0.72)	2.53 (0.74)	6.16 (3.15) (medium risk)

Chemical and microbial	Stockbreeding, agricultural activities within 50 m of the borehole	2.19 (0.60)	2.60 (0.69)	5.96 (2.71) (low risk)

Microbial	Rainstorms or floods affect the borehole area	2.20 (0.76)	2.38 (0.80)	5.72 (3.41) (low risk)

Chemical, microbial, and physical	Waste disposal site or landfill within 300 m of the borehole	2.07 (0.66)	2.29 (0.76)	5.15 (2.99) (low risk)

Risk level = likelihood ∗ Severity.

**Table 4:** Assessment of hazardous events at brackish desalination plants by seven raters.

Hazard	Hazardous event	Likelihood avg (SD)	Severity avg (SD)	Risk level avg (SD)
Microbial	Low level of disinfection below the recommended limit	3.27 (1.00)	3.60 (1.05)	12.71 (6.55) (high risk)

Chemical and microbial	The power outages affect the efficiency of the desalination process	3.25 (3.23)	2.24 (2.23)	10.04 (9.99) (high risk)

Chemical and microbial	Desalinated water is mixed with nondesalinated water	2.49 (1.34)	2.49 (1.38)	7.95 (7.32) (medium risk)

Physical, chemical, and microbial	The cap of the desalinated water tank is absent or partially opened	1.97 (1.07)	2.27 (1.28)	5.76 (5.79) (low risk)

Microbial	Water discontinuity or stored for an extended period inside the tank	2.04 (0.94)	2.25 (1.11)	5.57 (4.87) (low risk)

Physical, chemical, and microbial	Presence of foreign objects inside of desalinated water storage tank	1.88 (0.89)	2.23 (1.25)	5.23 (4.79) (low risk)

Microbial	The discharge pipes and hoses touch the ground	1.95 (1.07)	2.05 (1.19)	5.22 (5.71) (low risk)

Physical and microbial	Air vents unscreened allowing ingress of insects	2.49 (1.07)	1.83 (0.75)	5.21 (3.90) (low risk)

Chemical, physical, and microbial	The desalination plant is unlicensed and nonmonitored by the Ministry of Health	2.01 (1.60)	1.60 (0.87)	4.54 (5.64) (low risk)

Chemical and microbial	Excessive algal formations in filters due to irregular backwashing of filters	1.71 (1.21)	1.76 (1.25)	4.47 (6.46) (low risk)

Microbial	Desalination plant's fence or roof cover inadequate, which are allowing animals or birds entry	1.72 (0.82)	1.91 (1.07)	4.11 (4.26) (low risk)

Chemical	The desalinated water storage tank made of corrosive metal	1.69 (1.50)	1.40 (0.90)	3.68 (5.90) (low risk)

Chemical and microbial	Human excreta, sewage collection within 50 m of the desalinated water storage tanks	1.44 (1.14)	1.50 (1.27)	3.60 (6.76) (low risk)

Chemical, physical, and microbial	The drainage hole at the bottom of the tank is not available	1.83 (1.23)	1.48 (0.75)	3.53 (4.30) (low risk)

Microbial	There is leakage between desalination points and desalinated water storage tank	1.71 (0.81)	1.68 (0.78)	3.42 (3.01) (low risk)

Microbial	Spilled water forms stagnant ponding within 10 m of the desalinated water storage tanks	1.71 (0.81)	1.63 (0.71)	3.32 (2.90) (low risk)

Microbial	The desalinated water storage tank made of opaque plastic	1.42 (1.13)	1.41 (1.09)	3.23 (6.10) (low risk)

Chemical and microbial	The water storage tank used to store other liquids other than desalinated water	1.44 (0.93)	1.43 (0.92)	2.78 (4.09) (low risk)

Chemical	Accidental spillage of diesel within 100 m of the desalinated water storage tanks	1.38 (1.07)	1.26 (0.73)	2.51 (4.53) (low risk)

Chemical	Overdosing of treatment chemicals (such as chlorine)	1.06 (0.31)	1.06 (0.31)	1.23 (1.17) (low risk)

Risk level = likelihood ∗ Severity.

**Table 5:** Assessment of hazardous events in the tanker trucks by seven raters.

Hazard	Hazardous event	Likelihood avg (SD)	Severity avg (SD)	Risk level avg (SD)
Microbial	Low level of disinfection below the recommended limit	3.77 (0.80)	3.91 (0.85)	15.35 (6.01) (very high risk)

Chemical and microbial	Excessive algal formations inside the tanker truck tank	3.23 (1.07)	3.43 (1.06)	12.13 (7.27) (high risk)

Microbial	There is water leakage from the tanker reservoir or its pipes	3.79 (0.83)	2.85 (0.39)	10.98 (3.24) (high risk)

Chemical, physical, and microbial	The tanker truck is unlicensed and nonmonitored by the Ministry of Health	3.19 (0.86)	2.76 (0.48)	8.96 (3.31) (medium risk)

Chemical, physical, and microbial	The drainage tap at the bottom of the tank does not exist	2.86 (1.16)	2.35 (0.89)	7.59 (4.58) (medium risk)

Chemical and microbial	Desalinated water is mixed with nondesalinated water	2.35 (0.85)	2.56 (1.06)	6.87 (4.84) (medium risk)

Chemical, physical, and microbial	Presence of foreign objects inside of tanker truck tank	2.10 (0.87)	2.37 (1.08)	5.82 (4.43) (low risk)

Microbial	Water discontinuity or stored for an extended period inside the tanker truck tank	2.04 (0.85)	2.09 (0.95)	4.91 (3.97) (low risk)

Microbial	The parking garage of the tanker truck not sanitary	2.24 (1.03)	1.84 (0.86)	4.87 (4.43) (low risk)

Chemical and microbial	The delivery nozzle, pump, or hoses are dirty or in poor condition	2.00 (0.73)	2.12 (0.72)	4.70 (2.88) (low risk)

Chemical, physical, and microbial	The discharge pipes and hoses touch the ground	2.20 (0.91)	1.85 (0.82)	4.69 (3.74) (low risk)

Chemical, physical, and microbial	The cap of the tanker truck tank is absent or partially opened	1.60 (0.83)	1.88 (1.14)	3.90 (4.47) (low risk)

Chemical, physical, and microbial	The tanker truck engine exhaust and road contaminants affect the water quality	1.62 (0.86)	1.80 (1.05)	3.75 (4.33) (low risk)

Chemical, physical, and microbial	The overflow pipe is unclean or without protective mesh	1.68 (0.94)	1.67 (0.85)	3.47 (3.35) (low risk)

Chemical	The tanker truck desalinated water storage tank made of corrosive metal	1.53 (1.12)	1.49 (1.05)	3.44 (5.26) (low risk)

Chemical and microbial	The tanker truck tank used to store other liquids other than desalinated water	1.56 (0.65)	1.31 (0.57)	2.31 (2.07) (low risk)

Microbial	The tanker truck desalinated water storage tank made of opaque plastic	1.20 (0.74)	1.13 (0.50)	1.72 (2.73) (low risk)

Chemical	A high level of overdosing of treatment chemicals (such as chlorine)	1.08 (0.26)	1.03 (0.17)	1.14 (0.55) (low risk)

Risk level = likelihood ∗ Severity.

**Table 6:** Assessment of hazardous events in the drinking water at houses by seven raters.

Hazard	Hazardous event	Likelihood avg (SD)	Severity avg (SD)	Risk level avg (SD)
Microbial	Low level of disinfection below the recommended limit	3.87 (0.98)	4.02 (0.92)	16.38 (6.93) (very high risk)

Chemical and microbial	Excessive algal formations inside of desalinated water storage tank	3.62 (1.05)	3.82 (1.07)	14.82 (6.96) (high risk)

Microbial	The drinking water tank is not periodically washed with chlorine and soap	2.07 (1.25)	2.32 (1.42)	6.48 (7.24) (medium risk)

Chemical, physical, and microbial	Hand washing is not practiced before filling the drinking water storage tank	2.33 (1.25)	1.74 (0.78)	4.91 (4.47) (low risk)

Microbial	The desalinated water storage tank made of opaque plastic	2.27 (1.37)	1.47 (0.68)	4.15 (4.34) (low risk)

Microbial	Water discontinuity or stored for an extended period inside the desalinated water storage tank	2.27 (0.88)	1.47 (0.70)	3.74 (3.18) (low risk)

Microbial	The area around drinking water storage tank is unclean and has an animal or birds access	1.39 (0.93)	1.56 (2.23)	3.28 (5.51) (low risk)

Chemical	The desalinated water storage tank made of corrosive metal	1.37 (0.92)	1.51 (1.23)	3.17 (5.49) (low risk)

Chemical and microbial	Desalinated water is mixed with nondesalinated water	1.46 (0.80)	1.58 (1.01)	3.08 (4.02) (low risk)

Microbial	Drinking water storage tank is sited close to a toilet	1.33 (0.89)	1.42 (1.10)	2.85 (5.28) (low risk)

Chemical, physical, and microbial	Presence of foreign objects inside of desalinated water storage tank	1.38 (0.73)	1.50 (0.95)	2.74 (3.65) (low risk)

Chemical, physical, and microbial	The cap of the desalinated water tank is absent or partially opened	1.38 (0.73)	1.46 (0.86)	2.61 (3.34) (low risk)

Microbial	Children get drinking water directly from the tank tap by their mouths	1.30 (0.88)	1.31 (0.89)	2.46 (4.70) (low risk)

Microbial	The drinking water storage tank is leaking	1.19 (0.67)	1.25 (0.83)	2.03 (3.74) (low risk)

Microbial	The drinking water storage container sited at ground level	1.20 (0.74)	1.15 (0.58)	1.80 (3.23) (low risk)

Microbial	Spilled water accumulates around the drinking water storage tank	1.18 (0.65)	1.14 (0.49)	1.66 (2.47) (low risk)

Chemical and microbial	The water storage tank used to store other liquids other than desalinated water	1.09 (0.46)	1.14 (0.70)	1.56 (2.86) (low risk)

Chemical	A high level of overdosing of treatment chemicals (such as chlorine)	1.01 (0.09)	1.01 (0.12)	1.03 (0.27) (low risk)

Risk level = likelihood ∗ Severity

## Data Availability

The data used to support the findings of this study are available from the corresponding author upon request.

## Abbreviations

DWSSs: Drinking water supply systems
WHO: World health organization
WSP: Water safety plan
DALYs: Disability-adjusted life years
GDWQ: Guidelines for drinking water quality.
